# Survey data on heterogeneity in consumers’ food choice in eastern India

**DOI:** 10.1016/j.dib.2021.107148

**Published:** 2021-05-14

**Authors:** Jhoanne Ynion, Marie Claire Custodio, Arindam Samaddar, Suva Kanta Mohanty, Rosa Paula Cuevas, Anindita Ray (Chakravarti), Matty Demont

**Affiliations:** a International Rice Research Institute, Los Baños, Laguna, Philippines; b Kalinga Institute of Industrial Technology, KIIT School of Management, Bhubaneswar, India; c Institute of Rural Management Anand (IRMA), Anand, Gujrat, India; d Department of Food & Nutrition, Maharani Kasiswari College, University of Calcutta, India

**Keywords:** Gastronomic system, Food system, Food choice, Nutrition, Consumers, Eastern India

## Abstract

A consumer survey was conducted in eastern India in 2017 to understand the heterogeneity of consumers’ food choice. Face-to-face interviews were conducted among urban and rural consumers from low- and middle-income households in Odisha and West Bengal, eastern India, using a structured questionnaire. A multi-stage sampling procedure was implemented with stratified random sampling as the first stage and systematic sampling as the second stage. The survey data comprise responses from 501 respondents who have active involvement in grocery purchase decision-making and/or in meal planning or cooking for the household. The survey generated a dataset that was used to unravel five sources of heterogeneity (5Ws) in gastronomic systems that affect consumers' diets: (i) socioeconomic characteristics of the target population *(who)*; (ii) food environments *(where)*; (iii) eating occasions *(when)*; (iv) consumed dishes *(what)*; and (v) ingredient attributes and consumer attitudes towards food *(why)*. The approach and analyses are elaborated in the article “Unraveling heterogeneity of consumers’ food choice: Implications for nutrition interventions in eastern India”. Data from the survey can be further used to design behavioral experiments and interactive food choice tablet applications to elicit behavioral intentions in food choice.

## Specifications Table

**Table:** 

Subject	Social Sciences (General)
Specific subject area	Behavioral drivers of food choice; Gastronomic systems [1], [2]
Type of data	Raw and cleaned data files
How data were acquired	The data were obtained through a survey in selected urban and rural districts in Odisha and West Bengal in eastern India. Dish consumption and frequency of consumption of each dish were elicited through a list of pre-determined dishes consumed during different eating occasions (i.e., breakfast, morning snacks, lunch, afternoon snacks, dinner, and special occasions). Respondents identified the dishes their household consumes in a typical month and estimated the frequency of consumption using a scale. The attitude towards food quality attributes, food purchase, and food access was also acquired through a 5-point Likert scale indicating importance. The India Human Development Survey-II (IHDS-II) 2011–2012 [3] was used as the reference for income and area classifications. Sampling weights were calculated based on the population share for each income and area classification following the equation used by Bairagi et al. [4].
Data format	Raw and cleaned
Parameters for data collection	The survey was conducted in the key urban consumption zones and four rural districts in each state. The capital cities (i.e., Bhubaneswar in Odisha and Kolkata in West Bengal) were considered to represent the urban consumption zones. The rural districts were purposively selected based on nutritional status [5], population size [6], and geographic distribution. Some of the key criteria for a household to be interviewed are active involvement in grocery purchase decision-making or cooking/meal preparation/meal planning for the household and belonging to the low- and middle-income class.
Description of data collection	Data collection was done door-to-door through face-to-face interviews using a structured pen-and-paper questionnaire in Odisha and West Bengal in eastern India. A multi-stage sampling procedure was implemented with stratified random sampling and systematic sampling as the first and second stages in selecting a household. The research team developed a structured questionnaire and used show cards to elicit information from the respondents. The interviews were conducted by trained enumerators, and the completed questionnaires were checked by field supervisors for completeness and consistency.
Data source location	Country: India City/Town/Region: West Bengal: Kolkata, South 24 Parganas, Bankura, Murshidabad, and Jalpaiguri; Odisha: Bhubaneswar, Ganjam, Mayurbhanj, Kalahandi, and Bargarh
Data accessibility	Repository name: Harvard Dataverse Data identification number: 10.7910/DVN/IRSFPY Direct URL to data: https://doi.org/10.7910/DVN/IRSFPY
Related research article	Custodio, M.C., Ynion, J., Samaddar, A., Cuevas, R.P., Mohanty, S.K., Ray (Chakravarti), A., Demont, M. Unraveling heterogeneity of consumers’ food choice: Implications for nutrition interventions in eastern India, Glob. Food Sec. 28, 100497 (2021). https://doi.org/10.1016/j.gfs.2021.100497[7].

## Value of the Data

• Data from the consumer survey reveal the sources of heterogeneity in gastronomic systems which affect consumers’ food choice and diets of low- and middle-income households in eastern India [1], [2]. The gastronomic system features three levels—occasions, dishes, and ingredients—as possible entry-points for nutrition interventions. Accounting for heterogeneity of food choices can help policy makers and nutritionists develop more targeted nutrition interventions, which can aid in the development of “planetary health diets” in various contexts [8], [9].
• Insights about the sources of consumers’ heterogeneity of food choices and possible nutrition interventions through the gastronomic system and the food environment can help policy makers and nutritionists design segmented nutrition interventions strategies to improve diets and nutrition of urban and rural communities in eastern India [7].
• Data from the consumer survey can be used to design behavioral experiments and interactive food choice tablet applications to elicit behavioral intentions in food choice. Following the toolkit developed by Cuevas et al. [1], a three-stage mixed methods research approach can be used to capture diversity and drivers of food choice of a target population and identify entry points for nutrition interventions. Our consumer survey focuses on Stage 2 and validates and quantifies the preceding qualitative findings in Stage 1. Insights from the survey were then used to help design the subsequent behavioral experiment (Stage 3) to test the impact of a nutrition intervention on households’ food choice planning [10].
• The consumer survey captures five sources of heterogeneity (5Ws) in gastronomic systems that affect diets: (i) socio-demographic characteristics of the target population *(who);* (ii) food environments *(where)*; (iii) eating occasions *(when)*; (iv) consumed dishes *(what)*; and (v) ingredient attributes and consumer attitudes towards food *(why)* [7].

## 1 Data Description

The dataset includes 501 respondents from low- and middle-income households in key urban consumption zones and rural districts in eastern Indian states (i.e., Odisha and West Bengal) [9]. The data comprise responses from males and females aged 18 to 60 years old who have active involvement in grocery purchase decision-making and/or in cooking/meal preparation/meal planning for the household. In capturing the five sources of heterogeneity (5Ws) in gastronomic systems that affect diets, the survey data contains the following: (i) socio-demographic profile, involvement in purchase of food items and meal preparation/meal planning, household composition, and health status *(who)*; (ii) consumers’ physical access to various food products and perceptions on the promotional aspect of food *(where)*; (iii) information on eating occasions (i.e., breakfast, morning snacks, lunch, afternoon snacks, dinner, and special occasions), and frequency of consumption during these occasions *(when)*; (iv) dishes typically consumed in a month and frequency of consumption *(what)*; and (v) perception towards food quality attributes *(why)* [7], [9].

## 2 Experimental Design, Materials and Methods

### 2.1 Sampling approach

Through face-to-face interviews, a door-to-door survey was conducted from November to December 2017 in urban and rural districts of Odisha and West Bengal [7]. The geographic scope includes key urban consumption zones (i.e., the capital cities: Bhubaneswar in Odisha and Kolkata in West Bengal) and four rural districts for each state. The rural districts were purposively selected based on nutritional status (i.e., proportion of the population that is under- or over-nourished, based on body mass index) [5], population size based on the 2011 population census [6], and their geographic spread. A multi-stage sampling procedure was developed and followed in selecting the districts, primary sampling units, households, and qualified household members. The first sampling stage implemented stratified random sampling where city or rural districts were stratified into geographical zones (i.e., north, south, east, and west). Primary sampling units (PSUs) were randomly selected in each zone. The PSUs in the cities were streets, while PSUs in the rural districts were the villages within the 30-kilometer radius from the town center's border, a parameter set for practical purposes in implementing the survey in the rural districts. The second stage applied systematic sampling. The randomly selected PSUs served as the starting point following the right-hand rule from a starting point with a sampling interval of three households.

Screening questions in the questionnaire were then used to identify possible qualified household members. One of the key qualifying criteria for respondent selection is active involvement in grocery purchase decision-making or active involvement in cooking/meal preparation/meal planning for the household. In cases where more than one household member qualified to be interviewed, the person who had the most recent birthday was selected to be interviewed. Another qualifying criterion is the monthly household income. Our study's target population is urban and rural low- and middle-income households in eastern India [7]. The low-income range was defined as INR 15,000 and below and INR 7,000 and below for households in the urban and rural districts, respectively. The middle-income range was defined as INR 15,001–85,000 and INR 7,001–50,000 for households in the urban and rural districts, respectively. The India Human Development Survey-II (IHDS-II) 2011–2012 [3] was used to reference the income ranges and classifications.

### 2.2 Data collection

A pen-and-paper structured questionnaire, accompanied by show cards, was used to elicit information from the respondents. The questionnaire was translated into local languages, namely, Oriya for Odisha and Bengali for West Bengal. To ensure that the context is captured in the questionnaire, back-translations were conducted. Prior fieldwork, mock interviews were conducted to familiarize the interviewers with the questionnaire and train them to deliver the questions. Pilot interviews were conducted to test the flow and logic of the questionnaire and run through the designed sampling approach. Key information regarding consumers’ consumption behavior was collected, particularly the dishes consumed during different daily eating occasions and the corresponding frequency of consumption of each dish to assess the heterogeneity of households’ food choice. Consumers’ attitudes towards food quality attributes of dishes and association with specific dishes was assessed by attitudinal statements towards food for the daily eating occasions. Questions related to purchase and food access were included to gain insights into how consumers interact with their food environment.

### 2.3 Sampling weights

Over-representing certain population segments such as income classes is possible in a survey despite implementing a random sampling procedure [11]. In this study, sampling weights were used in the analysis to reduce sampling bias and to account for possible distorted population distribution of low- and middle-income households in urban and rural districts of each state. In calculating the sampling weights, we referred to the India Human Development Survey-II (IHDS-II) 2011–2012 [3] for the population distribution across income class (i.e., low- and middle-income) and area classification (i.e., urban cities and rural districts) in Odisha and West Bengal. Following the equation used by Bairagi et al. [4], the weighting factors were calculated as $w_{mn}=\frac{s_{mn}^{p}}{s_{mn}^{s}},\;$where $w_{mn}\;$is the weight for the *m*-th income class and *n*-th area classification; $s_{mn}^{p}\;$is the share of the population in *m*-th income class and *n*-th area classification; and $s_{mn}^{s}$ is the share of sampled survey respondents in *m*-th income class and *n*-th area classification.

### 2.4 Data analyses and software

Consumption behavior and attitudes were mainly assessed through diet diversity and through Exploratory Factor and Cluster analyses, respectively. Diet diversity and the average frequency of consumption were computed based on each household's frequency of consumption of dishes they consume in a typical month [7]. For every eating occasion (i.e., breakfast, morning snacks, lunch, afternoon snacks, dinner), each respondent was asked to identify the dishes their household consumes in a typical month and describe the frequency of consumption using a scale. A midpoint value was then assigned as follows: everyday = 28; 4–6 times per week = 20; 2–3 times per week = 10; once a week = 4; 2–3 times per month = 2.5; once a month = 1. Diet diversity (i.e., number of food groups in each eating occasion and frequency of consumption) was computed by classifying each dish into (i) starch-based, (ii) fruits/fruit-based, (iii) non-vegetarian (which includes meat, egg, fish, and prawns), (iv) dairy-based, (v) pulses, and (vi) vegetables based on the main ingredient of the dish. To generate the proportion of frequency of consumption of a food group on a given occasion ($p_{ik}$), the total frequency of consumption of dishes for each food group was computed and was divided by the total frequency of consumption of dishes for all food groups consumed by a respondent using the following formula: $p_{ik}=\sum_{j}f_{ijk}/\sum_{k}\sum_{j}f_{ijk}\;$where $f_{ijk}$ is the frequency of consumption of dish *j* classified in the food group *k* of respondent *i*. The average share of frequency of consumption for each food group on a given occasion was then computed using this formula: $\bar{p_{k}}=\sum_{i}p_{ik}/n\;$where $p_{ik}$ is the proportion of frequency of consumption of food group *k* of respondent *i*. The monthly average frequency of consumption of each dish was computed based on the sum of the frequency of consumption and the number of mentions.

The survey resulted in variations of dish names that led to sparsity in frequency data. To reduce sparsity, dishes that were highly similar but were referred to differently were grouped under the same name (e.g., “Egg poncho” and “Dim bhaja” were reassigned as “Egg omelet”). Data of dish consumption frequencies were stored in a relational database generated using MySQL Workbench (Version 8.0.13) and was accessed in Python (Version 3.6.6) through the SQLAlchemy object-relational mapper (Version 1.2.7). Co-occurrence matrices of dish consumption frequencies were developed using Python's pandas (Version 0.23.0) [12] and NumPy (Version 1.17.4) [13] libraries. These co-occurrence matrices were visualized as heat maps using the Seaborn (Version 0.9.0) [9], [14] and the Matplotlib (Version 2.2.2) [15] packages.

Spearman rank correlation, a non-parametric test, was used to test the strength and direction of the association between the frequency of purchase and distance of store type. Consumers’ attitude towards food was assessed through Exploratory Factor Analysis (EFA) [16]. The respondents were asked to evaluate 11 predefined statements relating to food quality attributes for each eating occasion (i.e., breakfast, morning snacks, lunch, afternoon snacks, and dinner) using a 5-point importance scale indicating a rating of 5 = Extremely important, 4 = Very important, 3 = Neutral, 2 = Of little importance, and 1 = Not at all important [7]. These statements stem from the focus group discussions conducted prior to the survey [1]. Before EFA, reliability tests were performed among statements for each eating occasion (Cronbach's α above 0.8) [16]. A seven-factor solution with 60% of total variance explained was generated using the Principal Component Method and varimax rotation. Items with loading values above the cut-off of 0.50 were considered on the label selected to represent each factor [16]. Negative factor scores are interpreted to be negatively related to the factor. Consumer segmentation [7] based on the themes derived from the EFA was done through non-hierarchical K-means clustering [16]. Using the seven factors from the EFA as cluster seeds, a three-cluster solution was selected to represent the consumer segments. The characteristics of the typical case for each cluster are indicated by cluster centers, computed as the mean for each variable within each cluster. One-way ANOVA was used to assess significant differences between the means of the three clusters. Statistical packages (i.e., IBM SPSS Statistics ver. 21 and StataSE ver. 14) were used in generating descriptive statistics and statistical analyses.

## Ethics Statement

The door-to-door survey with households was organized under the “Behavioral drivers of food choice in eastern India” project. The survey questionnaire obtained ethics approval from the International Rice Research Institute's (IRRI) Institutional Research Ethics Committee (IREC 18–001). Before the start of each interview, the respondents were informed that the survey is for research purposes, their participation is voluntary, and that all personal information will be kept confidential. The respondents gave verbal consent to be interviewed.

## CRediT Author Statement

**Jhoanne Ynion:** Writing – original draft, Writing – review & editing, Investigation, Software, Data curtion, Formal analysis, Project administration; **Marie Claire Custodio:** Writing – original draft, Writing – review & editing, Methodology, Investigation, Software, Formal analysis, Project administration; **Arindam Samaddar:** Methodology, Validation, Project administration, Resources; **Suva Kanta Mohanty:** Methodology, Validation, Resources; **Rosa Paula Cuevas:** Validation, Resources, Software, Data curtion, Formal analysis; **Anindita Ray:** Validation, Resources; **Matty Demont:** Conceptualization, Methodology, Validation, Writing review & editing, Supervision, Funding acquisition.

## Declaration of Competing Interest

The authors declare that they have no known competing financial interests or personal relationships which have, or could be perceived to have, influenced the work reported in this article.
